# Editorial: Visual code: From the retina to the brain

**DOI:** 10.3389/fncel.2022.1018229

**Published:** 2022-09-14

**Authors:** Tamás Kovács-Öller, Karin Dedek, Daniel Hillier

**Affiliations:** ^1^University of Pécs, Pécs, Hungary; ^2^Animal Navigation / Neurosensorics Group, Institute for Biology and Environmental Sciences, University of Oldenburg, Oldenburg, Germany; ^3^Hungarian Academy of Sciences, Budapest, Hungary; ^4^Faculty of Information Technology and Bionics, Pázmány Péter Catholic University, Budapest, Hungary; ^5^German Primate Center, Göttingen, Germany

**Keywords:** vision, retina, visual processing, visual cortex, lateral geniculate nucleus

The mammalian visual system begins in the eye, where the retina transforms the multi-dimensional visual field into distinct information channels that are then broadcasted to a range of subcortical nuclei. The visual subcortical nuclei, most prominently the dorsal lateral geniculate nucleus, further relay visual information to the primary and higher visual cortical areas to construct and update our internal model of the visual environment. The majority of the subcortical nuclei that do not project to the visual cortex drive or modulate eye movements, circadian entrainment, and other light-induced functions.

How the visual circuitry is constructed and how it achieves, and how this machinery achieves the feats of mammalian vision, are still active areas of research. Starting from the seminal works that identified the physiological properties of neuronal networks in the retina (Barlow, 1953; Kuffler, 1953; Barlow and Hill, 1963) and the primary visual cortex (Hubel and Wiesel, 1959) penned by the pioneers of visual neuroscience, generations of scientists have remained passionate and have kept designing the most creative experiments and tools to understand how the mammalian visual system works.

When putting research findings into a larger context, it is always important to be aware of the choice of model species. This is of particular importance when we ask how vision works. The visual systems of rodents, carnivores, and primates differ substantially, due to the absence or presence of a high-acuity region in their retinas and the resulting adaptations in higher brain areas and capacity for eye movements.

In this Research Topic, eight papers sample the vast landscape of visual neuroscience, spanning from rodent to human vision, including various species such as mice, rats, cats, macaques, and humans. Three of them focus on the retina; one on a subcortical nucleus, the pulvinar; three on the primary visual cortex; and one on method development.

The first retina paper looks at the survival of intrinsically photosensitive retinal ganglion cells (ipRGCs), which have long been in the visual neuroscience spotlight (Panda et al., 2002). Abed et al. found that knocking out Tbr2 (T-box brain protein 2, or eomesodermin) in adult mice results in the loss of melanopsin expression in ipRGCs but does not lead to cell death or morphological changes. In addition, they show that Tbr2+ ganglion cells preferentially survive optic nerve crush, confirming the preferential ipRGC survival reported by Pérez de Sevilla Müller et al. (2014).

The second retina study, by Wang et al., demonstrates that even low-dose atropine, used in the treatment of myopia, can affect visual signal processing. This could have a direct effect on the vision of treated individuals without affecting the morphology of their retina.

The third contribution related to the retina, by Zhang et al., provides a demonstration that Optical Coherence Tomography imaging can segment layers of the inner plexiform layer in the human retina.

Subcortical visual processing is represented in this Research Topic by Cortes et al., who used coupled dynamical systems to model the cortico-pulvinar network. Their model predicts that the pulvinar has at least two functional response states: regular oscillatory activity or stable asynchronous spiking. Cortico-pulvinar projections from the primary visual cortex and area 21a (cat homolog of primate V4) can drive the switch between these functional pulvinar states. The article models the role and background of oscillations in the pulvinar and brings the field of biologically inspired artificial visual systems back into focus. These systems play an important role in putting the notion of the “Visual Code” into a formal computational framework.

Three papers focus on the visual cortex. Chan et al. probe into the mechanisms of binocular matching at the end of the critical period of visual development in rats. They provide a rich account of the physiological, morphological, and molecular changes that occur as part of a highly specific method for prolonging the closure of the critical period.

Carmi et al. compared seven different methods for extracting retinotopic maps from simulated and *in vivo* voltage-sensitive dye imaging data in rats and found two methods that far outperform the other methods when applied to simulated and imaging data.

Hu et al. investigated the effect of attentional modulation on the functional interaction of (direction-selective) simple and complex cells with non-overlapping receptive fields in the macaque primary visual cortex. They found that spatial attention affected foremost the interactions between simple and complex cells, and not the interactions within these cell classes. In particular, the authors found that increases in attention correlated with increases in the spike counts of simple and complex cell pairs and a decrease in Granger causality. These effects were affected by the cell's preferred directions.

Finally, Arvin et al. present a new Python-based software tool for fast and efficient real-time pupil tracking, with example data across species. Their open-source solution both serves the needs of basic science and can also be used as an inspiring educational tool, e.g., to demonstrate closed-loop control of pupil size.

The eight papers of this Research Topic delved into several key topics within the visual neuroscience field, but of course, not every major direction of research could be covered here. The reader may nevertheless appreciate that visual neuroscience offers a lot of exciting avenues for research. The insights gained from better understanding how vision works in model species and from modeling may also bring us closer, step by step, to understanding the visual system of the human brain.

## Author contributions

All authors listed have made a substantial, direct, and intellectual contribution to the work and approved it for publication.

## Funding

DH was supported by the Momentum LP2020-3/2020 and NKFIH FK18-129120 grants. KD acknowledges funding from the DFG (RTG 1885/2 Molecular Basis of Sensory Biology), the European Union under the action of ERA-NET NEURON (JTC2020: Rethealthsi), and financed by the German Federal Ministry of Education and Research (BMBF, 01EW2107).

## Conflict of interest

The authors declare that the research was conducted in the absence of any commercial or financial relationships that could be construed as a potential conflict of interest.

## Publisher's note

All claims expressed in this article are solely those of the authors and do not necessarily represent those of their affiliated organizations, or those of the publisher, the editors and the reviewers. Any product that may be evaluated in this article, or claim that may be made by its manufacturer, is not guaranteed or endorsed by the publisher.
